# Outer membrane protein size and LPS O-antigen define protective antibody targeting to the *Salmonella* surface

**DOI:** 10.1038/s41467-020-14655-9

**Published:** 2020-02-12

**Authors:** C. Coral Domínguez-Medina, Marisol Pérez-Toledo, Anna E. Schager, Jennifer L. Marshall, Charlotte N. Cook, Saeeda Bobat, Hyea Hwang, Byeong Jae Chun, Erin Logan, Jack A. Bryant, Will M. Channell, Faye C. Morris, Sian E. Jossi, Areej Alshayea, Amanda E. Rossiter, Paul A. Barrow, William G. Horsnell, Calman A. MacLennan, Ian R. Henderson, Jeremy H. Lakey, James C. Gumbart, Constantino López-Macías, Vassiliy N. Bavro, Adam F. Cunningham

**Affiliations:** 1 Institute of Immunology and Immunotherapy, University of Birmingham, Birmingham, B15 2TT UK; 2 Institute of Microbiology and Infection, University of Birmingham, Birmingham, B15 2TT UK; 3 Medical Research Unit on Immunochemistry, Specialties Hospital, National Medical Centre “Siglo XXI” Mexican Institute for Social Security, Mexico City, Mexico; 4 School of Materials Science and Engineering, Georgia Institute of Technology, Atlanta GA, 30332 USA; 5 Institute of Infectious Disease and Molecular Medicine, University of Cape Town, Anzio Road, Cape Town, Western Cape, 7925 South Africa; 6 School of Veterinary Medicine and Science, University of Nottingham, Sutton Bonington, Leicestershire LE12 5RD UK; 7 Jenner Institute, Nuffield Department of Medicine, Old Road Campus Research Building, Roosevelt Drive, University of Oxford, Oxford, OX3 7DQ UK; 8 Institute for Cell and Molecular Biosciences, University of Newcastle, Newcastle upon Tyne, NE2 4HH UK; 9 School of Physics, Georgia Institute of Technology, Atlanta, GA 30332 USA; 10 School of Life Sciences, University of Essex, Wivenhoe Park, Colchester, CO4 3SQ UK

**Keywords:** Biophysics, Bacterial infection, Protein vaccines, Bacteria, Structural biology

## Abstract

Lipopolysaccharide (LPS) O-antigen (O-Ag) is known to limit antibody binding to surface antigens, although the relationship between antibody, O-Ag and other outer-membrane antigens is poorly understood. Here we report, immunization with the trimeric porin OmpD from *Salmonella* Typhimurium (STmOmpD) protects against infection. Atomistic molecular dynamics simulations indicate this is because OmpD trimers generate footprints within the O-Ag layer sufficiently sized for a single IgG Fab to access. While STmOmpD differs from its orthologue in *S*. Enteritidis (SEn) by a single amino-acid residue, immunization with STmOmpD confers minimal protection to SEn. This is due to the OmpD-O-Ag interplay restricting IgG binding, with the pairing of OmpD with its native O-Ag being essential for optimal protection after immunization. Thus, both the chemical and physical structure of O-Ag are key for the presentation of specific epitopes within proteinaceous surface-antigens. This enhances combinatorial antigenic diversity in Gram-negative bacteria, while reducing associated fitness costs.

## Abstract (web summary)

The O-antigen of LPS is known to limit the binding of antibody to bacterial surface antigens. Here the AUs show that the chemical and physical structure of the O-antigen are central factors in limiting the exposure of surface antigens to antibodies during *Salmonella* infection, thus defining their protective qualities.

## Introduction

Antibodies to the Gram-negative bacterial surface, induced after natural infection or vaccination, save millions of lives each year, and typically function before disease develops, reducing the need to use antibiotics and the development of antimicrobial resistance. After natural infection, the greatest level of protection is typically against the infecting strain, yet this is unsatisfactory for protection after vaccination, where wide-ranging immunity to multiple, related pathogens is clearly desirable. Achieving this is not always straightforward as shown by the nature of extant vaccines against Gram-negative bacteria. The only licensed single-antigen vaccines against diseases caused by such bacteria are based on capsular polysaccharides. When capsules are not utilized, or not available, then vaccines become increasingly antigenically complex, containing a mixture of purified antigens, such as the Bexsero vaccine to meningococcus serotype B or the acellular pertussis vaccine^1,2^. Even more antigenically complex are vaccines based on attenuated organisms, which offer the greatest breadth of antigen inclusion, but with little empirical rationalization as to which antigens are protective. This highlights our limited understanding of how protective antibodies interact with the bacterial surface.

To understand how antibodies interact with the Gram-negative bacterial surface, it is essential to consider the relationship between antibodies, LPS, and protein antigens. LPS consists of a conserved lipid A moiety and core oligosaccharide. In addition, it typically has a polysaccharide side chain that varies between different bacterial serotypes. This polysaccharide O-antigen (O-Ag) made up of varying numbers of repeats, sometimes > 100 in number, restricts access of molecules to the bacterial surface^3^. The consequence is that the millions of molecules of LPS found in each cell provide a formidable barrier to limit antibody access to the bacterial surface. Furthermore, LPS O-Ag can show significant structural variation even between closely related bacteria. For instance, different serovars of *Salmonella* express different O-Ag, such that *Salmonella* Typhimurium (STm) is partly defined by their possession of the O:4 antigen, whereas *S*. Enteritidis (SEn) is partly defined by the O:9 antigen.

In sub-Saharan Africa, invasive nontyphoidal salmonellosis (iNTS) is primarily caused by STm and SEn^4–7^. Antibodies to these strains strongly correlate with protection against invasive disease, and because of the devastating impact of iNTS there is an urgent need for a vaccine^8^. Achieving this would be greatly aided by understanding why some antigens are protective and others are not. Two frequently recognized cell-surface proteins are OmpA and OmpD. OmpA is a monomeric protein that is a common target of antibody after infection in mice and humans, and OmpD is found in the cell envelope in trimeric form^9^. Both antigens are highly conserved across Gram-negative bacteria, particularly within the same genus, meaning that an antibody to such a protein from one serovar may cross-protect against another. Previously, using a number of model systems, we had identified that IgG antibodies to the porin protein OmpD can offer cell-dependent or complement-dependent protection^10,11^. Unexpectedly, not all IgG isotypes offer equivalent protection, and this correlated to the level of access of antibodies to bind OmpD in the presence of O-Ag^12^. This suggests that O-Ag influences the level of binding of IgG to the bacterial surface. In this study, we tested this concept by examining the capacity of antibody to OmpD from STm (STmOmpD) to cross-protect against SEn, which contains a near-identical OmpD that differs by a single amino acid. Our findings suggest that how small changes in proteinaceous surface antigens of Gram-negative bacteria can combine with O-Ag to maximize antigen diversity within a single species, and have implications for generating vaccines offering multi-serovar protection.

## Results

### STmOmpD induces protective antibody responses

**Fig. 1:**
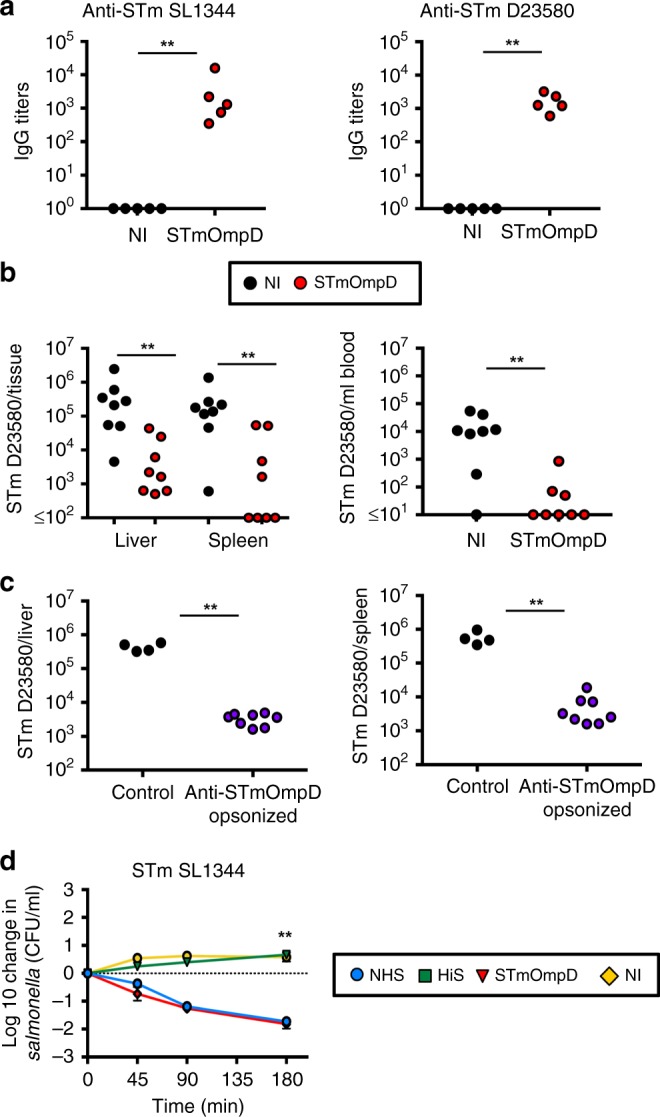
STmOmpD induces antibody that can impair infection against STm. **a** WT mice were primed and boosted i.p. with 20 μg of STmOmpD at days 0, 14, and 28, and sera obtained at day 35. Anti-STm (D23580 and SL1344) IgG was assessed by ELISA. Each point represents titers from an individual mouse, and is representative of three repeat experiments. **b** Bacterial numbers in the liver, spleen, and blood of nonimmunized (NI) and WT mice immunized twice (days 0 and 14) with 20 μg of STmOmpD and challenged with 1 × 10^5^ STm D23580 for 24 h. Each point represents bacterial numbers in one mouse. **c** Mice were infected i.p. with 5 × 10^5^ of either STm SL3261 or bacteria previously opsonized with STmOmpD antibodies. Bacteria numbers were quantified at day 3 from the spleen and liver. Data are representative of two independent experiments. **d** Serum bactericidal assays using STm SL1344 with sera from NI mice or mice immunized as in **a**, supplemented with human sera as a complement source with anti-STm-specific antibodies depleted. Normal human serum (NHS, blue) and heat-inactivated human serum (HiS, green) were used as controls. Negative values correspond with a decrease in viable STm compared with starting normals. Samples were taken at minutes 45, 90, and 180 using a starting concentration of 10^6^ CFU/ml. Sera from NI (yellow) and STmOmpD immune (red) WT mice were tested. Data are representative of two different experiments and represent the mean of 5–6 mice sera used. **P *≤* 0.01 assessed by two-tailed Mann–Whitney *U* test.

Wild-type (WT) mice were immunized twice with 20 μg of purified STmOmpD (Supplementary Fig. 1a and ref. ^12^) in the absence of additional adjuvant. The induced anti-STmOmpD IgG bound to the surface of two different isolates of STm: the model laboratory strain SL1344 and a representative invasive African isolate of STm, D23580, which was originally isolated from an African patient^13^ (Fig. 1a). WT mice immunized twice with 20 μg of STmOmpD and challenged intraperitoneally (i.p.) with STm D23580 for 24 h had a ≥ 100-fold median reduction in their bacterial burdens in the liver and spleen, and a near-10,000-fold reduction in the median bacterial numbers in the blood (Fig. 1b). Similar results were observed for the laboratory strain SL1344 (Supplementary Fig. 1b). To assess whether anti-STmOmpD antibody alone was sufficient to promote bacterial killing, bacteria were opsonized with complement-inactived anti-STmOmpD or control sera prior to infection. This showed that opsonization with anti-STmOmpD sera resulted in an ~100-fold reduction in bacterial numbers post infection (Fig. 1c). We then assessed if anti-STmOmpD sera could kill bacteria in a well-established serum bactericidal assay (SBA), which uses human sera depleted of anti-STm antibodies, as a source of complement^14^. In these experiments, anti-STmOmpD antibodies enhanced complement-dependent killing, whereas sera from control mice did not (Fig. 1d). Purified OmpD had low levels of co-purifying LPS (typical levels of LPS of ≤ 2 endotoxin units (EU) per 10 μg of porin by limulus assay), but some contaminating LPS, and the immunodominant protective O-Ag, could remain. To control for antibodies generated to residual O-Ag present in the OmpD being responsible for these effects, we immunized mice with OmpD purified from a triple-mutant strain of STm that lacks OmpF, OmpC, and O-Ag. Mice immunized with this OmpD exhibited similar levels of protection (Supplementary Fig. 1c). Therefore, anti-STmOmpD antibodies can reduce bacterial burdens in the liver, spleen, and blood after infection with laboratory and clinical strains of STm.

### STmOmpD immunization confers minimal cross-protection to SEn

**Fig. 2:**
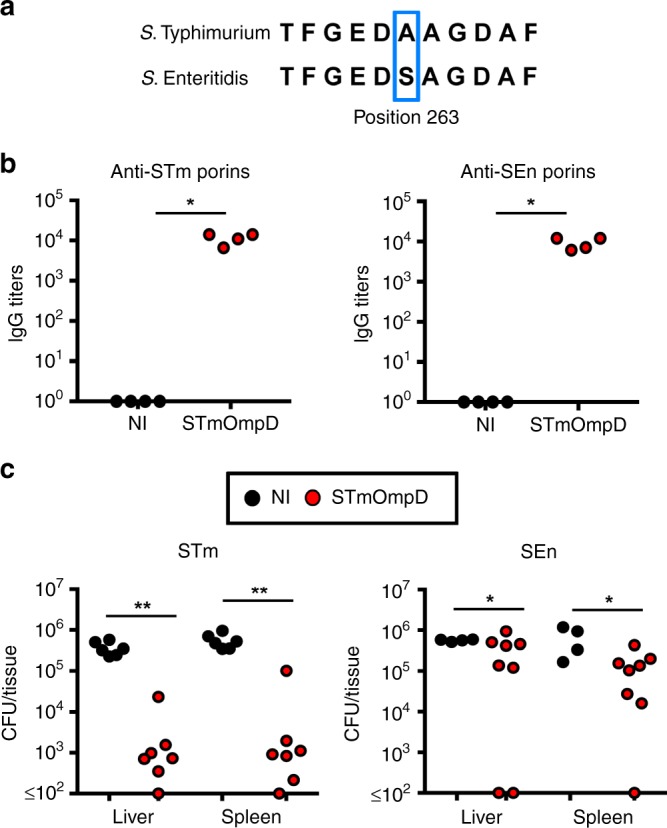
Antibodies to STmOmpD offer limited cross-protection to SEn. **a** Alignment of OmpD sequences from STm and SEn showing the site where an alanine-to-serine substitution is observed at amino acid 263 of the whole sequence. **b** Serum IgG titers to STm and SEn porins from WT NI mice and mice immunized with STmOmpD as in Fig. 1a. Each point represents the titers from one mouse. Data representative of two experiments. **c** NI mice and mice immunized twice with 20 μg of STmOmpD were challenged with 5 × 10^5^ *aroA*^−^ STm or SEn, and bacterial numbers per organ enumerated 3 days later. Each point represents the titers from one mouse. Data are representative of two individual experiments. *P *≤* 0.05, **P *≤* 0.01 assessed by two-tailed Mann–Whitney *U* test.

The two most commonly identified iNTS isolates found in sub-Saharan Africa are STm and SEn in which OmpD differs by a single amino acid, a substitution of an alanine in STmOmpD at position 263 to a serine in SEn (Fig. 2a; Supplementary Fig. 2). Moreover, anti-STmOmpD antibodies had similar levels of binding to purified porins from STm and SEn, indicating that there are multiple shared epitopes present between these proteins (Fig. 2b). Therefore, it was anticipated that immunization with STmOmpD would confer cross-protection against SEn, enabling its use within a multivalent vaccine. Mice were immunized twice with 20 μg of STmOmpD before challenge with STm or SEn. Immunization with STmOmpD induced statistically significant protection in both groups. However, while there was a near-100-fold reduction in immunized mice challenged with STm, the reduction in bacterial numbers in SEn-challenged mice was less than fivefold (Fig. 2c). Therefore, a single amino-acid variation associates with the loss of protection afforded by immunization with STmOmpD.

### Loss of O-Ag enhances efficacy of protection against SEn

**Fig. 3:**
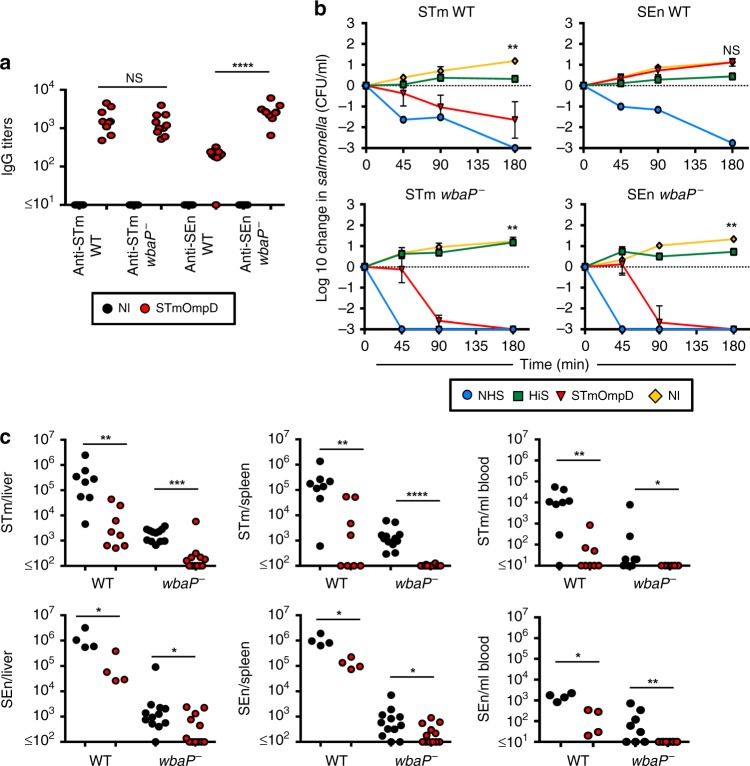
Loss of O-Ag from SEn enhances protection by anti-STmOmpD antibodies. **a** IgG titers to STm and SEn WT and O-Ag-deficient mutants in sera from NI mice and mice immunized with STmOmpD. **b** Serum bactericidal assays using STm D23580, SEn D24954, and STm and SEn *wbaP*^−^ mutants with sera from NI mice or mice immunized as in Fig. 1a, supplemented with human sera as a complement source with anti-STm-specific antibodies depleted. Normal human serum (NHS, blue) and heat-inactivated human serum (HiS, green) were used as controls. Negative values correspond with a decrease in viable STm compared with starting normals. Samples were taken at minutes 45, 90, and 180 using a starting concentration of 10^6^ CFU/ml. Sera from NI (yellow) and STmOmpD immune (red) WT mice were tested. **c** NI mice and mice immunized twice with 20 μg of STmOmpD were challenged with 1 × 10^5^ of STm or SEn (WT or *wbaP*^−^) for 24 h, and bacterial burdens determined from the spleen, liver, and blood. Each point represents the titers from one mouse. Data are from three different experiments. *P *≤* 0.05, **P *≤* 0.01, ***P *≤* 0.001, ****P *≤* 0.0001, NS = nonsignificant assessed by two-tailed Mann–Whitney *U* test.

The significantly diminished cross-protection to SEn after immunization with STmOmpD was unexpected. One interpretation of the reduced protection to SEn after immunization with STmOmpD is that the A263S variation is within an immunodominant epitope that is most readily accessible to IgG, and that other conserved epitopes are less accessible. To determine if LPS O-Ag impaired binding of anti-STmOmpD IgG to SEn, plates were coated with intact STm or SEn, or mutants that lack O-Ag (*wbaP*^−^). Binding of anti-STmOmpD IgG to O-Ag-expressing STm or O-Ag-deficient STm was similar, consistent with the antibody having a solely proteinaceous target (Fig. 3a). In contrast, when the coating antigen used was whole WT SEn, then antibody binding was dramatically lower than that observed for the different STm strains (Fig. 3a). However, binding of anti-STmOmpD IgG to O-Ag-deficient SEn was comparable with STm. This suggests that additional, conserved, epitopes became accessible to anti-STmOmpD IgG when O-Ag was absent. We then examined whether this enhanced binding of antibody to O-Ag-deficient SEn altered the bactericidal activity of anti-STmOmpD antibodies. Anti-STmOmpD antibody was bactericidal for STm, but not SEn (Fig. 3b). However, anti-STmOmpD serum could promote killing of O-Ag-deficient STm and O-Ag-deficient SEn bacteria. Consistent with this, in most mice immunized with STmOmpD, O-Ag-deficient SEn was not detectable at all (Fig. 3c). These experiments suggest that most IgG that binds OmpD on O-Ag-expressing bacteria targets a limited number of variable proteinaceous epitopes that include A263S, whereas O-Ag occludes binding to the larger number of epitopes conserved between the two proteins.

### OmpD trimer size allows IgG access through the LPS barrier

**Fig. 4:**
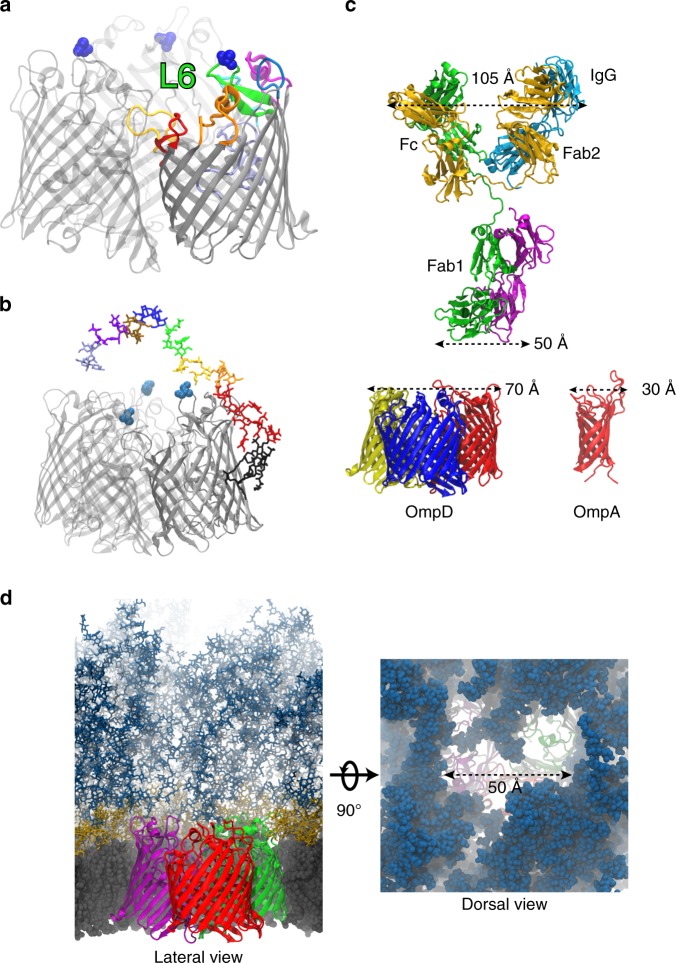
Acessibility of OmpD epitopes is determined by the LPS component. OmpD structure and the location of the A263S polymorphism in the context of interaction with the LPS layer. **a** OmpD trimer (homology model) with the location of the A263S variation (blue spacefill representation) on the tip of the extracellular loop 6 (labeled L6, green in protomer 1). The other extracellular loops in this protomer are shown in different colors consistent with their representation in Supplementary Figs. S2 and S6. **b** Snapshot of the full atomistic MD simulation of the outer membrane showing an isolated OmpD trimer with a single LPS molecule. LPS elements are colored as follows: Lipid A—black; core sugars in red; O-Ag repeats in rainbow colors. **c** Size comparison of a murine IgG1 molecule (based on PDB 1IGY) to the OmpD trimer and OmpA. Top shows an IgG1 molecule. The heavy chains are colored in green and gold, while the light chains are in cyan and purple, respectively. Dimensions of various parts are indicated in Å. Bottom left, an OmpD trimer (protomers shown in yellow, blue, and red) and bottom right an OmpA monomer (red). **d** A wider context of the MD system is shown from the membrane plane (left) and from the top (right). OmpD protomers are colored in purple, red, and green, and LPS elements are colored as follows: Lipid A and inner leaflet in gray, core sugars in gold, and O-Ag in dark blue. The dense network provided by the LPS O-Ag prevents access to the surface epitopes on OmpD, which is especially clear on the spacefilling top-view representation on the right, showing a typical access channel dimension of 50 Å.

We next assessed where the critical A263S variation was located within the protein, and found it mapped to the large surface-exposed loop 6 on the outer rim of the OmpD trimer. Creation of a homology model of the OmpD trimer revealed that A263 is at the tip of the loop most distal from the hydrophobic core of the membrane, and so potentially most accessible by antibody targeting OmpD (Fig. 4a). However, this site, on the outer rim of the OmpD trimer, is next to the surrounding LPS O-Ag, and thus antibody access to epitopes containing this residue may be restricted due to a combination of LPS O-Ag occlusion and the size of IgG antibodies. To examine the nature of antibody–OmpD interaction on the surface of the bacterium, we performed in silico analysis of the structures of OmpD, LPS, and antibody (Fig. 4b–d). Strikingly, the diameter of the OmpD trimer exceeds that of a Fab fragment (Fig. 4c: 70 Å for OmpD trimer and ~50 Å for Fab along the shortest dimension). This diameter of the OmpD trimer, and the associated LPS tunnel, is not influenced by the A263S variation. We then performed atomistic molecular dynamics (MD) simulations of the outer membrane, which included explicit modeling of the LPS from STm in two distinct random distributions of O-Ag length between 6 and 11 repeats, for 2 μs per system (Fig. 4d; Supplementary Fig. 3a). These simulations show that the LPS O-Ag provides a robust barrier to prevent access to the cell envelope, with each OmpD trimer tightly interacting with up to 15 molecules of LPS, greatly limiting the lateral mobility of OmpD in the membrane. While the chains of O-Ag sometimes partially occlude direct access in these simulations, their fluctuations occasionally reveal an open pathway to the surface (Fig. 4d). The prediction of accessibility of OmpD to IgG was reinforced by a 70-ns steered MD simulation of full IgG binding, which demonstrated that it can overcome the steric hindrance of the O-Ag layer to reach the OmpD loops (Supplementary Fig. 4). Furthermore, this simulation unequivocally demonstrated that at most a single Fab fragment could be accommodated within the footprint of the tunnel at a time. Therefore, the size of the OmpD footprint explains how IgG can gain access to the most-exposed surface loop epitopes of OmpD in the presence of O-Ag.

### O-Ag structure limits binding of anti-STmOmpD antibodies

**Fig. 5:**
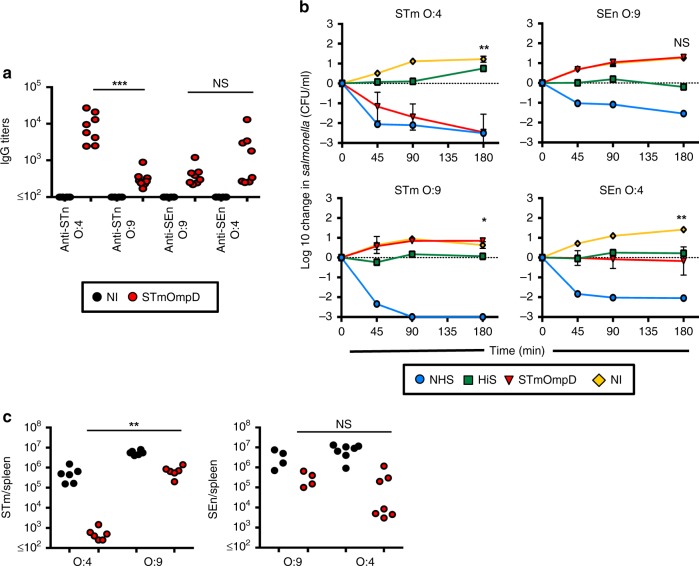
O-Ag structure and variability influence access of antibody to OmpD. **a** IgG titers to STm and SEn WT and LPS chimera strains were assessed using sera from NI mice and mice immunized with STmOmpD as in Fig. 1a. Each point represents the titers from one mouse. Representative of three experiments. **b** Serum bactericidal assays using STm D23580, SEn D24954, and STm O9 and SEn O4 chimera mutants with sera from nonimmunized mice (yellow) or mice immunized with STmOmpD (red) as in Fig. 1a, supplemented with human sera as a complement source with anti-STm-specific antibodies depleted. Normal human serum (NHS, blue) and heat-inactivated human serum (HiS, green) were used as controls. Negative values correspond with a decrease in viable STm compared with starting normals. Samples were taken at minutes 45, 90, and 180 using a starting concentration of 10^6^ CFU/ml. **c** Bacterial burdens from the spleens of NI and mice immunized twice with 20 μg of STmOmpD infected for 24 h with 1 × 10^5^ of STm or SEn WT and O-Ag chimeras. Data are representative of a minimum of two individual experiments. *P *≤* 0.05, **P *≤* 0.01, ***P *≤* 0.001, NS = nonsignificant assessed by two-tailed Mann–Whitney *U* test.

The previous experiments suggest a role for O-Ag in restricting access to epitopes on OmpD. The O4 and O9 O-Ag expressed by STm and SEn differ in their use of the sugars acetylabequose and tyvelose within their O-Ag, respectively (Supplementary Fig. 3b). Moreover, there is a strong correlation between the A263S polymorphism and O-Ag usage by *Salmonella* in groups B and D of the Kaufmann–White scheme (Supplementary Fig. 2). Therefore, we examined whether different O-Ag can influence access to epitopes on OmpD. To do this, we examined the level of binding of anti-STmOmpD to STm, SEn, and to chimera strains of STm and SEn engineered to express O9 and O4, respectively^15^. As expected, strong binding of anti-STmOmpD antibody to WT STm was detected, but antibody binding to WT SEn and both chimeric strains was markedly reduced (Fig. 5a). Furthermore, anti-STmOmpD antibody was only efficient at promoting the killing of WT STm in a SBA (Fig. 5b), or after challenge of mice immunized with STmOmpD (Fig. 5c). Moreover, after challenge of STmOmpD-immunized mice with WT OmpD-expressing STm, there was an approximately tenfold median reduction in bacterial numbers, compared with a 3–4 median-fold reduction in bacterial numbers after challenge with SEn or STm and SEn mutants that express the reciprocal OmpD gene (i.e., STm expressing OmpD 263S and SEn OmpD 263 A; Supplementary Fig. 5). In combination, these results support the conclusion that matched native O-Ag and OmpD are needed for optimal protection. As mentioned above, OmpD generates similarly sized footprints and corresponding tunnel apertures in the LPS layer of both serovars, despite the different O-Ag structures between STm and SEn. However, we did observe a difference in the interactions of extracellular loops 5, 6, and 8 of OmpD with LPS and O-Ag in the STm and SEn simulations. The average contact area between these loops and LPS was higher in both SEn systems compared with the corresponding STm systems (Supplementary Fig. 6). Despite a difference of just one sugar in the O-Ag of SEn (O4, tyvelose) and that of STm (O9, acetylabequose) (Supplementary Fig. 3b), the MD results suggest that this could account for the former being more flexible than the latter (Supplementary Fig. 7). These results highlight that O-Ag offers more than a simple steric barrier to antibody, and that specific interactions between the O-Ag and the protein component influence the physical presentation of the epitope, impacting its availability even when the antibody can access the antigen.

### Antibodies to monomeric OmpA are not protective

**Fig. 6:**
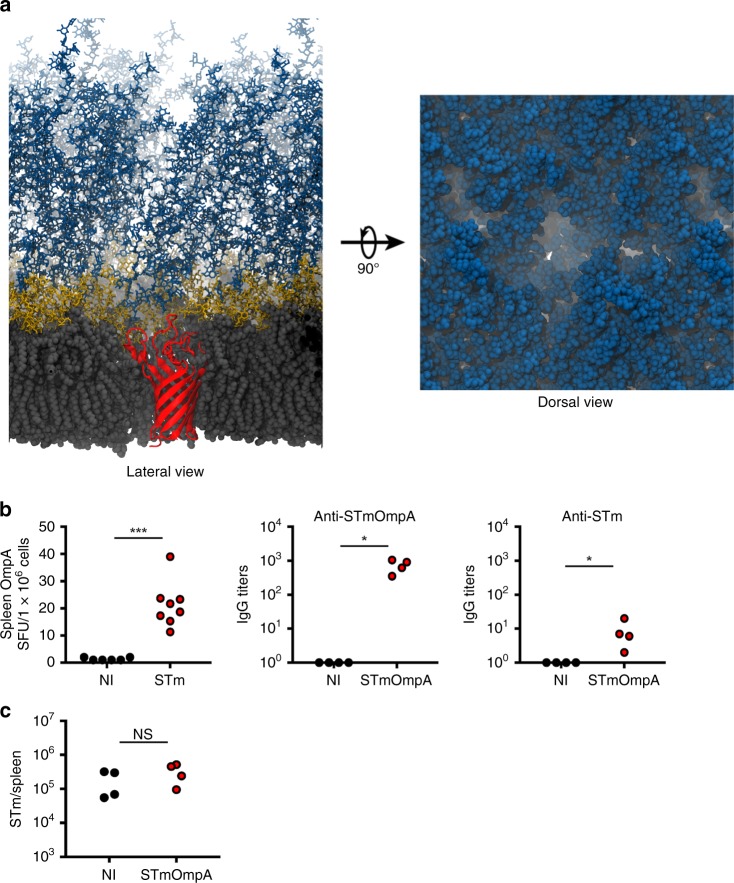
Anti-OmpA antibodies are not protective against infection. **a** A snapshot from the OmpA (red) after equilibration in the SEn outer membrane (membrane colored as in Fig. 4c) viewed from the membrane plane (left) and extracellular side (right) indicates that it will not be accessible to IgG due to LPS occlusion. **b** (Left) WT mice were challenged with 5 × 10^5^ STm SL3261 for 5 days, and the frequency of antibody-secreting cells (ASC) in the spleen assessed by ELISPOT. (Right) Anti-OmpA and anti-STm IgG titers of WT mice immunized twice with 20 μg of alum-precipitated OmpA were determined. Each point represents the titers from one mouse. Representative of two experiments. **c** Splenic bacterial burdens of WT NI mice and mice immunized with 20 μg of alum-precipitated OmpA mice challenged with 5 × 10^5^ STm for 4 days. Similar results seen at other time points up to 7 days post challenge. Each point represents the titers from one mouse. Data are representative of two independent experiments. *P *≤* 0.05, ***P *≤* 0.001, NS = nonsignificant assessed by two-tailed Mann–Whitney *U* test.

If the reason that OmpD induces protective antibodies is due to the aperture size generated in the cell envelope by the trimer protein, enabling access by antibody, then it could be expected that antibodies to other cell wall antigens that generate smaller tunnels would not be protective. OmpA has a similar molecular weight as an OmpD monomer (35.4 kDa vs. 37.6 kDa, respectively) (Fig. 4c). Unlike OmpD, OmpA does not typically form oligomers in the outer membrane, and induces specific antibodies rapidly after natural infection in mice and humans^16^. Using the same structural approaches as before, we show that OmpA creates a footprint that has an aperture that is too small for a Fab fragment to access (Fig. 6a). Due to this occlusion, it is unlikely to be a target of protective antibodies. Consistent with there being less immunological pressure on this protein, we found that, unlike OmpD, the OmpA sequence is identical between STm and SEn isolates. We then purified OmpA to test the ability of antibodies to OmpA to protect against natural infection in mice. Purified OmpA was recognized rapidly by antibody-secreting cells induced by natural infection of mice with STm and after immunization of mice with purified OmpA (Fig. 6b). Nevertheless, antibody to purified OmpA bound only weakly to intact bacteria, and did not induce protective responses (Fig. 6b, c). Therefore, despite being a target of naturally induced antibodies, this protein in STm and SEn is not under immune pressure, and does not induce protective responses.

## Discussion

Antibodies to STmOmpD protect against infection with STm^12^. Since there is only a single amino-acid difference between OmpD from STm and SEn, and anti-STmOmpD antibodies cross-react with SEn, we hypothesized that immunization with STmOmpD would protect against both STm and SEn. Unexpectedly, the level of cross-protection, while statistically significant, was minimal in terms of fold reduction in bacterial numbers. We show that this lack of cross-protection is due to the complex relationship between LPS O-Ag and OmpD.

O-Ag provides steric hindrance of antibody binding to the bacterial surface. While the capacity of O-Ag to block antibody access is well described in the literature^17,18^, the relationship between O-Ag and other surface antigens is poorly understood. In the context of OmpD, O-Ag restricted access to conserved epitopes that are proximal to the membrane surface. Conformational dynamics of LPS influence the behavior and presentation of outer membrane proteins (OMPs)^19–21^, suggesting that surface antigens need to be considered in the context of their native environment. The LPS layer is one of the most complex membrane environments and is notoriously difficult to replicate in vitro. Up until recently, it was poorly understood, which prevented even in silico approaches. Our work has been enabled by recent breakthroughs in atomistic simulations, which now permit explicit modeling of realistic outer membranes^20,22–25^. Nevertheless, there are limitations to our modeling associated with our incomplete understanding of the Gram-negative bacterial surface. For instance, although O-Ag chain length and distribution have been studied in isolation, as has protein clustering^21,26^, its effect in vivo and in the context of antibody binding is unclear. Nevertheless, our modeling suggests that the tunnel aperture size that is generated by the OmpD trimer in the surrounding LPS layer is only sufficiently sized to accommodate binding of a single Fab, and suggests that two independent antibodies cannot bind an individual OmpD trimer concurrently. An important and surprising implication of our results is that despite only a single Fab being able to access the LPS channel, it appears to be sufficient to promote complement-dependent killing. This conclusion is consistent with the experimental evidence showing similar binding of anti-STmOmpD IgG to WT or O-Ag-deficient STm and that bacterial outer-membrane vesicle vaccines derived from bacteria lacking O-Ag can offer protection^27^. This could indicate that it is not necessary to have both arms of an antibody bound to a target antigen for it to be protective. Antibodies to core oligosaccharide can be protective^28^. In this study, it is not possible to rule out the possibility that antibodies generated to the minimal core antigen co-purified with OmpD made some contribution to the protection observed. Nevertheless, multiple lines of evidence suggest that, if indeed present, any such contribution is minimal. First, the levels of endotoxin in preparations were minimal as assessed by limulus assay (< 2 EU/10 μg OmpD). Second, mice are not protected after immunization with OmpD if they are infected with an OmpD-deficient bacterium^10^. Third, modification of the sequence of OmpD or the O-Ag expressed, but retaining the same core antigen, resulted in reduced protection, which is inconsistent with antibodies to core antigen accounting for the protection observed. Relatedly, the predicted footprint generated by a single OmpD trimer in the LPS layer is similar for STm and SEn, yet there is minimal cross-protection, which is suggestive that anti-core antibodies make a minimal contribution to the protection afforded by immunization with purified OmpD.

Our studies show that although anti-STmOmpD sera recognize multiple epitopes, in reality the presence of O-Ag translates to protection being afforded by antibody binding to a restricted number of epitopes that likely include the A263S polymorphism. This means that, in terms of protection, not all epitopes are equal. The protective potential of individual epitopes will be influenced by the footprint of the antigen in the LPS layer, which has to be sufficiently large to alleviate the occlusion by O-Ag and allow access to its epitopes placing some epitopes under stronger immunological pressure than others. This may explain the A263S variation observed in loop 6 of OmpD, and is supported by the cross-serovar conservation of OmpA. Our immunological data are consistent with this interpretation, as it shows that antibody to OmpA does not bind the bacterial surface nor is protective. At the same time, our modeling predicts that the OmpA-generated tunnel in the O-Ag is insufficiently sized to enable ready access by antibody. Indeed, most published studies have not found protection after immunization with OmpA^29,30^. In one exception, a study from Nikaido et al.^31^, protective monoclonal antibodies against OmpA bound to a portion of OmpA that is only surface exposed when OmpA adopts an atypical conformation that results in a much larger footprint on the cell surface. This is wholly consistent with the size of the tunnel being key for enabling antibody access for binding and protection. Sequence variation in the surface-exposed loops of other outer-membrane proteins, such as the monomeric proteins OmpX and OmpW, and the trimeric porin OmpC is similar to what we have described here. OmpX and OmpW are identical between STm and SEn, but there are differences in OmpC, which are conserved between STm and SEn isolates, respectively, and thus may reflect some association with O-Ag usage. In contrast, monomeric OMPs do not appear to be under selective pressure to change. These findings are consistent with the principles identified in this study and could be predicted based on our findings with OmpD and OmpA, correlating with the inability of the antibodies to penetrate the narrow footprint created by monomeric porins.

An additional level of complexity is added by the diversity of O-Ag structure, which can impede antibody binding to OmpD. We show that antibody to OmpD to be protective then the O-Ag must match the parent serovar of the OmpD. This dual requirement was unexpected, but shows how antigens can combine to enhance antigenic diversity. This was demonstrated most clearly with the challenge experiments using chimaeric strains, since the parent O-Ag and the A263S variation need to match for the antibody to bind. This could explain why for *Salmonella* within groups B and D of the Kauffmann–White scheme, there is a close correlation between a specific O-Ag and the polymorphism present in OmpD. Independently, modeling suggested that differential O-Ag usage has an impact on the self-association of O-chains, which may affect the nature of antibody interaction, and by extension, the accessibility of the surface epitopes. This observation adds another level of modulation, to the known capacity of LPS to stabilize antigen conformation^20,21^.

Since O-Ag usage varies between many Gram-negative bacteria, our finding on the importance of the footprint made by proteinaceous antigens and the relationship to immunological protection has significant implications for vaccine design. Subunit vaccines that protect against Gram-negative bacteria are typically based on the surface capsular polysaccharide. There is no single surface protein antigen subunit vaccine for Gram-negative bacteria and if a capsule antigen is not used in the vaccine, then they increase considerably in their complexity. One reason for this increased complexity may be the influence of LPS/LOS O-Ag on the accessibility of antibody to the component antigens, meaning that greater antigen coverage is needed to ensure protection. In contrast, since capsules are typically located on the outermost surface of the bacterium, it may mean there is less impediment to antibody binding and less need to include multiple antigens in a vaccine.

One benefit from the shielding offered by O-Ag is to reduce the immunological pressure to introduce base changes into a protein, which could have fitness costs for the bacterium. To achieve a single amino-acid change in OmpD requires as few as one nucleotide change. In contrast, to alter O-Ag usage requires significant changes in multiple genes involved in the biosynthetic pathway. Nevertheless, the occlusion of antibody binding to the bacterial surface by O-Ag reveals an intrinsic paradox as O-Ag itself a target of protective antibodies, yet is not under the same selective pressure within the same serovar. Although, the reasons for this paradox are unclear, this may reflect our incomplete understanding of the nature of antibody binding to the bacterium or be due more to wholly unrelated reasons such as evading attack by bacteriophages. Collectively, our results suggest that merging structural modeling and immunology can predict which antigens, and the epitopes within those antigens, may be protective and such a priori knowledge may aid in development of future vaccines against Gram-negative infections.

## Methods

### Bacterial strains, immunogens, mice, and immunizations

Invasive African isolates *S*. Typhimurium D23580 and *S*. Enteritidis D24954^8,13^, the virulent STm SL1344 laboratory strain and the attenuated STm SL3261 *aroA*^−^ and SEn P125109 *aroA*^−^ were used in experiments^32,33^. Rough *wbaP*^−^ STm D23580 and SEn P125109 mutants, and STm O9 and SEn O4 LPS O-Ag chimera strains were kindly provided by Dr Chris Coward (Cambridge)^15^. To generate STm D23580 or SEn D29540 with chromosomal mutations so that STm expressed an OmpD with a serine at amino-acid position 263, while SEn OmpD with an alanine at the same position, an established protocol was used^34^. Using this, the positive–negative selection cassette, encoding the kanamycin resistance marker and the rhamnose-inducible *relE* toxin (kan-P_*rhaB*_*-relE*), was amplified from pSLC217 using oligonucleotides containing the universal priming sites P1 and P2 with 50 bp of homology to the centre of the *ompD* gene at the 5′ end of each primer (ompDKOpSLC217Fwd: 5′-GGCCTGGTTGAAGGTCTGAACTTTGCCGCGCAGTACCAGGGCAAAAACGACCGTGACTGT GTAGGCTGGAGCTGCTTC-3′ and ompDKOpSLC217Rev: 5′-TCGTACTCATACGTTGCGGACAGACCGAAACCGTCGCCGTTAGACTCGTACGCGCCGTCCATATGAATATCCTCCTTAG-3′. The homology regions used were designed to insert the cassette into the middle of *ompD* (nt 540 from the translation start site) without replacing any of the *ompD* coding sequence. STm D23580 or SEn D29540 was transformed with the λ-RED encoding plasmid pSIM18 by electroporation, and transformants were selected by growth on LB agar supplemented with hygromycin (75 μg/ml) at 30 °C^35^. The *ompD* flanked kan-P_*rhaB*_*-relE* cassette was then recombined into the chromosome using established approaches^34^, except for the following modifications. Cells were grown initially at 30 °C for 2 h after which λ-RED expression, from pSIM18, was induced by temperature shift to 42 °C for 1 h before cells were made electrocompetent and transformed with the *ompD* flanked kan-P_*rhaB*_*-relE* cassette. Recombinants were selected by growth on LB agar containing kanamycin (50 μg/ml) at 37 °C. The presence of the kan-P_*rhaB*_*-relE* cassette was confirmed by PCR of the *ompD* chromosomal locus followed by agarose gel electrophoresis. The *ompD* gene was then amplified from STm D23580 or SEn D29540 and used in a second round of recombineering, as described above, in order to replace the kan-P_*rhaB*_*-relE* cassette disrupted *ompD* gene with the heterologous *ompD* gene. Recombinants were selected by growth on M9 agar supplemented with 0.2% rhamnose. The presence of the heterologous *ompD* gene in each strain was confirmed by PCR and sequencing of the *ompD* chromosomal locus (ompDfwd: 5′-ATGAAACTTAAGTTAGTGGCAGTG-3′ and ompDrev: 5′-TTAGAACTGGTAGTTCAGACCAACAG-3′). Strains were also checked for loss of the pSIM18 plasmid by patch plating onto LB agar supplemented with hygromycin. Wild-type (WT) C57BL/6 or CD1 mice between 6 and 12 weeks of age were used. Mice were obtained from HO Harlan OLAC Ltd. (Bicester, UK) and from the Animal Facility at the University of Cape Town (UCT). Mice were maintained under specific pathogen-free conditions. Animal experiments were performed with full local (University of Birmingham or University of Cape Town, Protocol 012-006) and national ethical approval (for the UK compliance with the Animals (Scientific Procedures) Act 1986 (project licences 30/2850 and P2E63AE7B). Mice were infected intraperitionally (i.p.) as described for each experiment. OMPs were purified by 2% (v/v) Triton X-100 extraction from bacterial pellets^36^. STmOmpD (from OmpC and OmpF-deficient STm SL1344 or an OmpC, OmpF, and wbaP-deficient mutant) and STm or SEn porins were purified by 2% (v/v) Triton X-100 extraction and repeated extraction with SDS and separation by fast protein liquid chromatography on a Sephacryl S-200 column before dialysis against PBS/0.1% (w/v) SDS in PBS^10,12,37^. The level of LPS detected in OmpD preparations by limulus assay was typically ≤ 2 EU / 10 μg OmpD. Mice were immunized with 20 μg of OmpD i.p. or PBS as described. For opsonization experiments, 5 × 10^5^ bacteria/ml were mixed for 30 min with individual heat-treated anti-STmOmpD sera or sera from nonimmunized mice, diluted 1:100. Mice were then infected with 5 × 10^5^ bacteria i.p. In each case, bacteria were cultured to confirm that the pre-opsonization of bacteria did not induce clumping or affect viability.

### Sequence analysis and homology modeling

The multiple sequence alignments (MSA) were prepared using MAFFT and NJ/UPGMA phylogeny algorithms as implemented in MAFFT v.7 server (https://mafft.cbrc.jp/)^38^, and Espript 3^39^ was used for visualization and structural annotations. For the purposes of homology modeling which was based on the sequence of OmpD from *Salmonella* LT2 (Uniprot ID: P37592 (OMPD_SALTY)), we employed I-TASSER^40^ with manual assignment of templates. We used OmpF from *E.coli* (1OPF.pdb)^41^, OmpC (5NUP:A.pdb)^42^, and Osmoporin (Ompk36) from *Klebsiella pneumoniae* (1OSM.pdb)^43^ as templates based on highest identity scores (63%, 64%, and 65% respectively), with trimerization docking, local manual refinement and structural superposition performed in Coot^44^. Figures were prepared using Pymol (The PyMOL Molecular Graphics System, Version 1.71 Schrödinger, LLC) unless otherwise specified.

### ELISA and serum bactericidal assays

Antibody specificity to STm and SEn porins, STmOmpD, and whole organisms was tested by ELISA^45^. Plates were coated with antigen at 5 μg/ml overnight. Mouse serum was added to wells with a starting dilution of 1:50, then serially diluted and the presence of bound antibody was detected with IgG-specific alkaline phosphatase (AP)-conjugated goat anti-mouse antibody (Southern Biotech, catalogue 1015-04, dilution 1:2000) and developed with Sigma FAST™ p-Nitrophenyl Phosphate tablets. Titers were calculated by identifying the dilution at which the signal reached a set OD_405_.

Serum bactericidal assays were performed^8^, using human serum was used as a complement source for murine antibodies^14^. Sera from mice immunized with STmOmpD were supplemented with antibody-depleted human serum (1:2–1:18 dilutions from mice sera were made to give a final volume of 45 μl). A volume of 5 μl of *Salmonella* in logarithmic (log) growth phase (1 × 10^7^ CFU/ml) per sample was added to a v-bottomed 96-wells plate (Sarstedt). Heat-inactivated human serum (HiS) was added in a 1:1 proportion with PBS as a negative control. Sera from nonimmunized mice were used as controls. Samples were incubated at 37 °C on a rocker plate with shaking at 20 rpm, and samples plated in triplicate on agar plates 45, 90, and 180 min after incubation. Growth or killing of the bacteria was represented graphically by transforming the data into log_10_.

### MD simulations

The OmpD trimer from STm or SEn was placed in a corresponding outer membrane model that included an outer leaflet of LPS molecules and an inner leaflet composed of 75% POPE and 25% POPG lipids, built using VMD^46^. The length of the O-Ag chain of each LPS molecule was randomly assigned to be between 6 and 11 repeating units. Because LPS diffusion is too slow to avoid bias due to the initial distribution in simulations^20,47^, two models with different random distributions were constructed for each serovar. An alternative approach is to use coarse-grained simulations, which can reach 1–2 orders-of-magnitude longer time scales^48–50^. Since each LPS molecule is negatively charged (−12), Mg^2+^ and Ca^2+^ ions were added to neutralize the system (236 and 52 for the A systems, respectively, and 241 and 47 for the B systems, respectively). Next, Na^+^ and Cl^−^ ions were added to bulk solvent to a concentration of 150 mM, resulting in a system size of ~300,000 atoms. Each of the four systems (two for STm and two for SEn) was run for 1 μs, followed by two 500-ns replicas that were used for analysis (2 μs of simulation for each of the four systems). The simulations were performed using NAMD^51^ and Amber16^52^ along with the CHARMM36 force field^53,54^ in the NPT ensemble at a temperature of 310 K and a pressure of 1 atm. A 2-fs time step was used for the first 250 ns, after which hydrogen mass repartitioning (HMR) was employed to reach a time step of 4 fs for the remainder of the simulations. HMR has recently been demonsrated to be applicable to membrane-protein systems with little to no effect on dynamics^55^. For the steered MD simulation, a murine IgG1 antibody (taken from PDB 1IGY) was added to the STm-B system and pulled towards the OmpD loops at 1.0 Å/ns for 70 ns.

Convergence of the simulations was judged by monitoring the membrane area and the RMSD of the OmpD trimer, both of which were stabilized within 1 μs in most cases (see Supplementary Figs. 8 and 9, respectively). The time scale required is similar to that observed in previous simulations of outer membranes^56,57^. Finally, although the LPS does not diffuse on the time scale of our simulations, the O-Ag have greater freedom of movement, as shown in Supplementary Fig. 7.

### Statistical analysis

Data were analyzed using GraphPad Prism Version 6.0. Differences in nonparametric data were determined with the Mann–Whitney *U* test. For SBA data, the Mann–Whitney *U* test was used to determine the difference in growth at 180 min between immune and non-immune mice sera.

### Reporting summary

Further information on research design is available in the Nature Research Reporting Summary linked to this article.

## Supplementary information

Supplementary Information

Reporting Summary

## Data Availability

The authors declare that all data supporting the findings of this study are available within the paper and its supplementary information files. The source data underlying Figs. 1a–d, 2b–c, 3a–c, 5a–c, 1b–c, and Supplementary Figs. 1a–c, and 5 are provided as a Source Data file. The whole datasets are available from the corresponding authors on reasonable request.

## Source Data

Source Data

## References

[1] Edwards KM (1995). Comparison of 13 acellular pertussis vaccines: overview and serologic response. Pediatrics.

[2] Gorringe AR, Pajon R (2012). Bexsero: a multicomponent vaccine for prevention of meningococcal disease. Hum. Vaccin. Immunother..

[3] Peterson AA, McGroarty EJ (1985). High-molecular-weight components in lipopolysaccharides of *Salmonella typhimurium*, *Salmonella minnesota*, and *Escherichia coli*. J. Bacteriol..

[4] MacLennan CA, Martin LB, Micoli F (2014). Vaccines against invasive Salmonella disease: current status and future directions. Hum. Vaccin. Immunother..

[5] Feasey NA, Dougan G, Kingsley RA, Heyderman RS, Gordon MA (2012). Invasive non-typhoidal salmonella disease: an emerging and neglected tropical disease in Africa. Lancet.

[6] de Jong HK, Parry CM, van der Poll T, Wiersinga WJ (2012). Host-pathogen interaction in invasive Salmonellosis. PLoS Pathog..

[7] Hohmann EL (2001). Nontyphoidal salmonellosis. Clin. Infect. Dis..

[8] MacLennan CA (2008). The neglected role of antibody in protection against bacteremia caused by nontyphoidal strains of Salmonella in African children. J. Clin. Investig..

[9] Nikaido H (2003). Molecular basis of bacterial outer membrane permeability revisited. Microbiol. Mol. Biol. Rev..

[10] Gil-Cruz C (2009). The porin OmpD from nontyphoidal Salmonella is a key target for a protective B1b cell antibody response. Proc. Natl Acad. Sci. USA.

[11] MacLennan CA (2010). Dysregulated humoral immunity to nontyphoidal Salmonella in HIV-infected African adults. Science.

[12] Zhang Y (2017). IgG1 is required for optimal protection after immunization with the purified porin OmpD from *Salmonella Typhimurium*. J. Immunol..

[13] Kingsley RA (2009). Epidemic multiple drug resistant *Salmonella Typhimurium* causing invasive disease in sub-Saharan Africa have a distinct genotype. Genome Res..

[14] Siggins MK (2011). Absent bactericidal activity of mouse serum against invasive African nontyphoidal Salmonella results from impaired complement function but not a lack of antibody. J. Immunol..

[15] Goh YS (2015). Monoclonal antibodies of a diverse isotype induced by an O-antigen glycoconjugate vaccine mediate in vitro and in vivo killing of African invasive nontyphoidal Salmonella. Infect. Immun..

[16] Lee SJ (2012). Identification of a common immune signature in murine and human systemic Salmonellosis. Proc. Natl Acad. Sci. USA.

[17] Bentley AT, Klebba PE (1988). Effect of lipopolysaccharide structure on reactivity of antiporin monoclonal antibodies with the bacterial cell surface. J. Bacteriol..

[18] van der Ley P, Kuipers O, Tommassen J, Lugtenberg B (1986). O-antigenic chains of lipopolysaccharide prevent binding of antibody molecules to an outer membrane pore protein in Enterobacteriaceae. Microb. Pathogenesis.

[19] Blasco P, Patel DS, Engstrom O, Im W, Widmalm G (2017). Conformational dynamics of the lipopolysaccharide from *Escherichia coli* O91 revealed by nuclear magnetic resonance spectroscopy and molecular simulations. Biochemistry.

[20] Balusek C, Gumbart JC (2016). Role of the native outer-membrane environment on the transporter BtuB. Biophysical J..

[21] Arunmanee W (2016). Gram-negative trimeric porins have specific LPS binding sites that are essential for porin biogenesis. Proc. Natl Acad. Sci. USA.

[22] Patel DS, Qi Y, Im W (2017). Modeling and simulation of bacterial outer membranes and interactions with membrane proteins. Curr. Opin. Struct. Biol..

[23] Matthias KA (2017). Heterogeneity in non-epitope loop sequence and outer membrane protein complexes alters antibody binding to the major porin protein PorB in serogroup B Neisseria meningitidis. Mol. Microbiol..

[24] Pavlova A, Hwang H, Lundquist K, Balusek C, Gumbart JC (2016). Living on the edge: simulations of bacterial outer-membrane proteins. Biochimica et. Biophysica Acta.

[25] Nascimento A, Pontes FJ, Lins RD, Soares TA (2014). Hydration, ionic valence and cross-linking propensities of cations determine the stability of lipopolysaccharide (LPS) membranes. Chem. Commun..

[26] Rassam P (2015). Supramolecular assemblies underpin turnover of outer membrane proteins in bacteria. Nature.

[27] Schager, A. E. et al. IgG responses to porins and lipopolysaccharide within an outer membrane-based vaccine against nontyphoidal salmonella develop at discordant rates. *mBio* **9**, e02379-17 (2018). 10.1128/mBio.02379-17 PMC5844998 29511082

[28] Cross AS (2014). Anti-endotoxin vaccines: back to the future. Virulence.

[29] Vuopio-Varkila J, Karvonen M, Saxen H (1988). Protective capacity of antibodies to outer-membrane components of *Escherichia coli* in a systemic mouse peritonitis model. J. Med. Microbiol..

[30] Okamura M (2012). Immunization with outer membrane protein A from Salmonella enterica serovar Enteritidis induces humoral immune response but no protection against homologous challenge in chickens. Poult. Sci..

[31] Singh SP, Williams YU, Miller S, Nikaido H (2003). The C-terminal domain of Salmonella enterica serovar typhimurium OmpA is an immunodominant antigen in mice but appears to be only partially exposed on the bacterial cell surface. Infect. Immun..

[32] Hoiseth SK, Stocker BA (1981). Aromatic-dependent Salmonella typhimurium are non-virulent and effective as live vaccines. Nature.

[33] Barrow PA, Lovell MA, Berchieri A (1991). The use of two live attenuated vaccines to immunize egg-laying hens against Salmonella enteritidis phage type 4. Avian Pathol.: J. W. V. P. A.

[34] Khetrapal V (2015). A set of powerful negative selection systems for unmodified Enterobacteriaceae. Nucleic Acids Res..

[35] Chan W (2007). A recombineering based approach for high-throughput conditional knockout targeting vector construction. Nucleic Acids Res..

[36] Henderson IR, Meehan M, Owen P (1997). Antigen 43, a phase-variable bipartite outer membrane protein, determines colony morphology and autoaggregation in Escherichia coli K-12. FEMS Microbiol. Lett..

[37] Salazar-Gonzalez RM (2004). Induction of cellular immune response and anti-Salmonella enterica serovar typhi bactericidal antibodies in healthy volunteers by immunization with a vaccine candidate against typhoid fever. Immunol. Lett..

[38] Katoh K, Misawa K, Kuma K, Miyata T (2002). MAFFT: a novel method for rapid multiple sequence alignment based on fast Fourier transform. Nucleic Acids Res..

[39] Gouet P, Robert X, Courcelle E (2003). ESPript/ENDscript: extracting and rendering sequence and 3D information from atomic structures of proteins. Nucleic Acids Res..

[40] Yang J (2015). The I-TASSER Suite: protein structure and function prediction. Nat. methods.

[41] Cowan SW (1995). The structure of OmpF porin in a tetragonal crystal form. Structure.

[42] Abellon-Ruiz J (2017). Structural basis for maintenance of bacterial outer membrane lipid asymmetry. Nat. Microbiol..

[43] Dutzler R (1999). Crystal structure and functional characterization of OmpK36, the osmoporin of Klebsiella pneumoniae. Structure.

[44] Emsley P, Lohkamp B, Scott WG, Cowtan K (2010). Features and development of Coot. Acta Crystallogr. Sect. D., Biol. Crystallogr..

[45] Cunningham AF (2007). Salmonella induces a switched antibody response without germinal centers that impedes the extracellular spread of infection. J. Immunol..

[46] Humphrey W, Dalke A, Schulten K (1996). VMD: visual molecular dynamics. J. Mol. Graph..

[47] Soares TA, Straatsma TP (2008). Assessment of the convergence of molecular dynamics simulations of lipopolysaccharide membranes. Mol. Simul..

[48] Van Oosten B, Harroun TA (2016). A MARTINI extension for *Pseudomonas aeruginosa* PAO1 lipopolysaccharide. J. Mol. Graph. Model..

[49] Jefferies D, Shearer J, Khalid S (2019). Role of O-antigen in response to mechanical stress of the *E. coli* outer membrane: insights from coarse-grained MD simulations. J. Phys. Chem. B.

[50] Shearer J, Jefferies D, Khalid S (2019). Outer membrane proteins OmpA, FhuA, OmpF, EstA, BtuB, and OmpX have unique lipopolysaccharide fingerprints. J. Chem. Theory Comput..

[51] Phillips JC (2005). Scalable molecular dynamics with NAMD. J. Comput. Chem..

[52] Case, D. A. *AMBER 2016* (University of California, San Francisco, 2016).

[53] Klauda JB (2010). Update of the CHARMM all-atom additive force field for lipids: validation on six lipid types. J. Phys. Chem. B.

[54] Best RB (2012). Optimization of the additive CHARMM all-atom protein force field targeting improved sampling of the backbone phi, psi and side-chain chi(1) and chi(2) dihedral angles. J. Chem. Theory Comput..

[55] Balusek Curtis, Hwang Hyea, Lau Chun Hon, Lundquist Karl, Hazel Anthony, Pavlova Anna, Lynch Diane L., Reggio Patricia H., Wang Yi, Gumbart James C. (2019). Accelerating Membrane Simulations with Hydrogen Mass Repartitioning. Journal of Chemical Theory and Computation.

[56] Kirschner KN, Lins RD, Maass A, Soares TA (2012). A glycam-based force field for simulations of lipopolysaccharide membranes: parametrization and validation. J. Chem. Theory Comput..

[57] Wu EL (2014). E. coli outer membrane and interactions with OmpLA. Biophysical J..

