# Supplementary material for: Outer membrane protein size and LPS O-antigen define protective antibody targeting to the Salmonella surface
Source: Nat Commun. 2020 Feb 12;11:851. doi: 10.1038/s41467-020-14655-9 (PMC7015928; doi:10.1038/s41467-020-14655-9)
Supplement: Supplementary file 1 — Supplementary Information [file 41467_2020_14655_MOESM1_ESM.pdf]

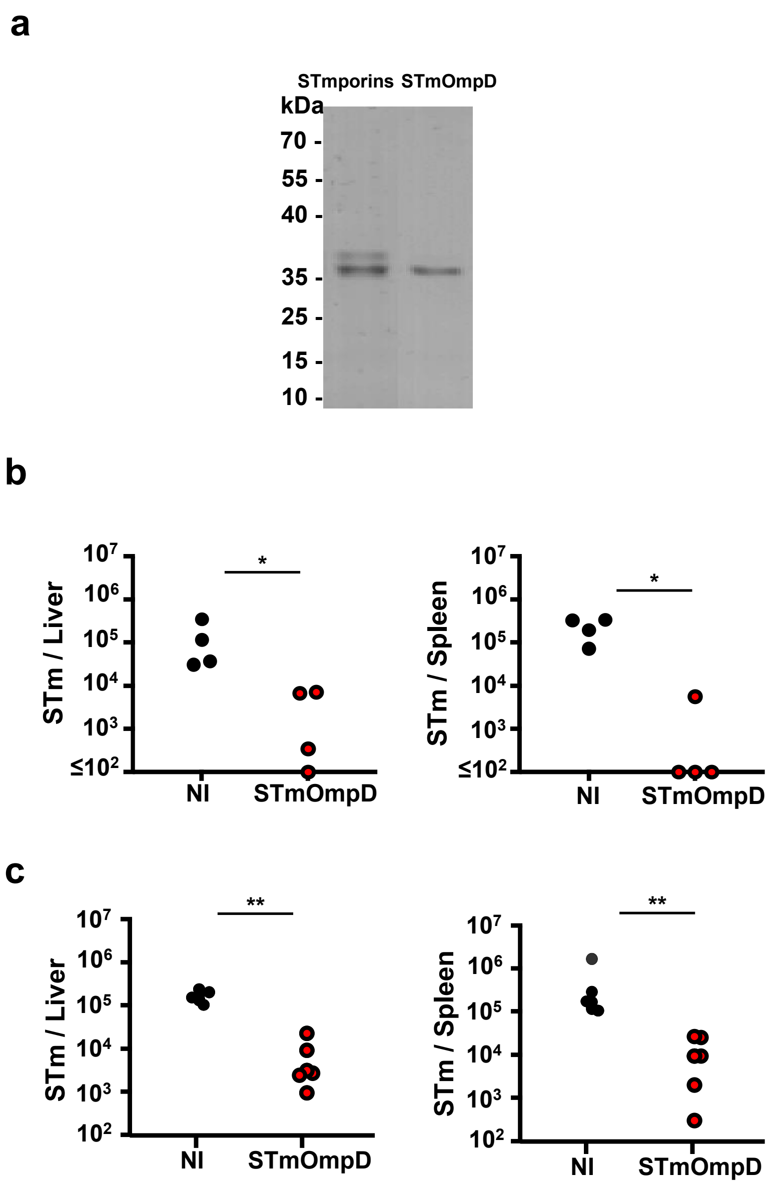

Supplementary Fig 1. STmOmpD protects against STm SL1344. **a** Coomassie blue-stained preparations of purified STm-porins (OmpF, OmpC and OmpD) and STmOmpD in a 15% SDS PAGE where samples were adjusted to a concentration of 1.1 µg; each band represents the molecular weight (kDa) of each of the proteins. **b** Bacterial numbers in the livers and spleens of NI mice and mice immunized twice with 20 µg STmOmpD on days 0 and 14 and challenged with STm SL1344 for 24 hours. **c** Bacterial numbers in the livers and spleens of NI mice and mice immunized twice with 20 µg STmOmpD purified from STm lacking OmpC, OmpF and wbaP, on days 0 and 14 and challenged with STm SL3261 for 24 hours. * = $P \leq 0.05$, ** = $P \leq 0.01$ assessed by 2-tailed Mann-Whitney U-test.

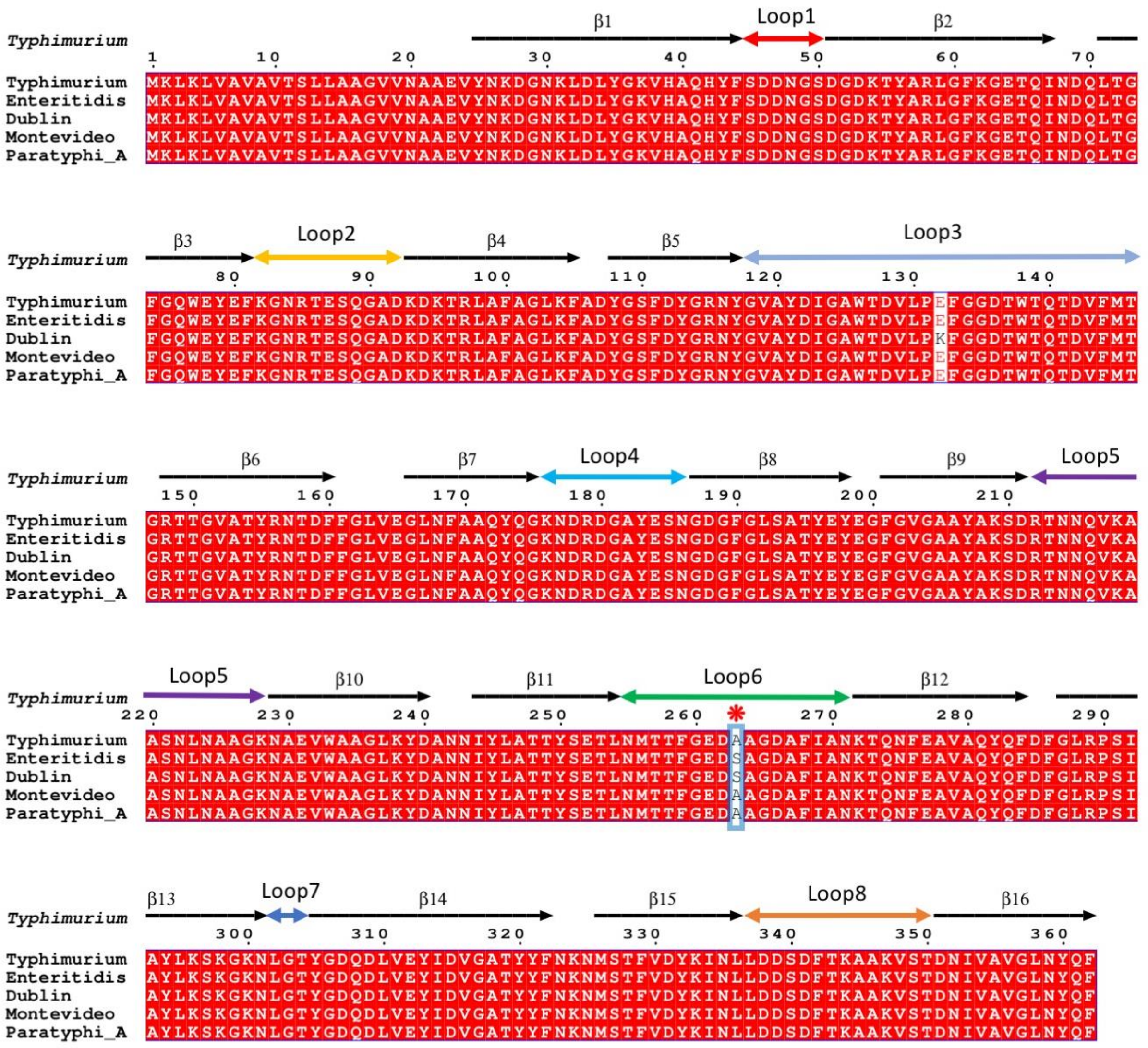

Supplementary Fig. 2. OmpD is highly conserved between in *Salmonella*. OmpD sequence representative of *Salmonella* serovars from different groups of the Kauffman-White classification scheme are aligned. *S*. Typhimurium (GenBank: CBW17597.1) is group B; *S*. Enteritidis (GenBank: EPI73535.1) is group D; *S*. Dublin (GenBank: EMR54474.1) is group D; *S*. Montevideo (GenBank: AHW09554.1) is group C and *S*. Paratyphi (UniProtKB/Swiss-Prot: Q5PHY0.1) is group A. The predicted topology diagram of the OmpD from *S.* Typhimurium, based on the homology model discussed in the text, is shown above its sequence, with loops and beta-strands forming the barrel numbered. The location of the A263S variation is indicated with a box and a star in loop 6.

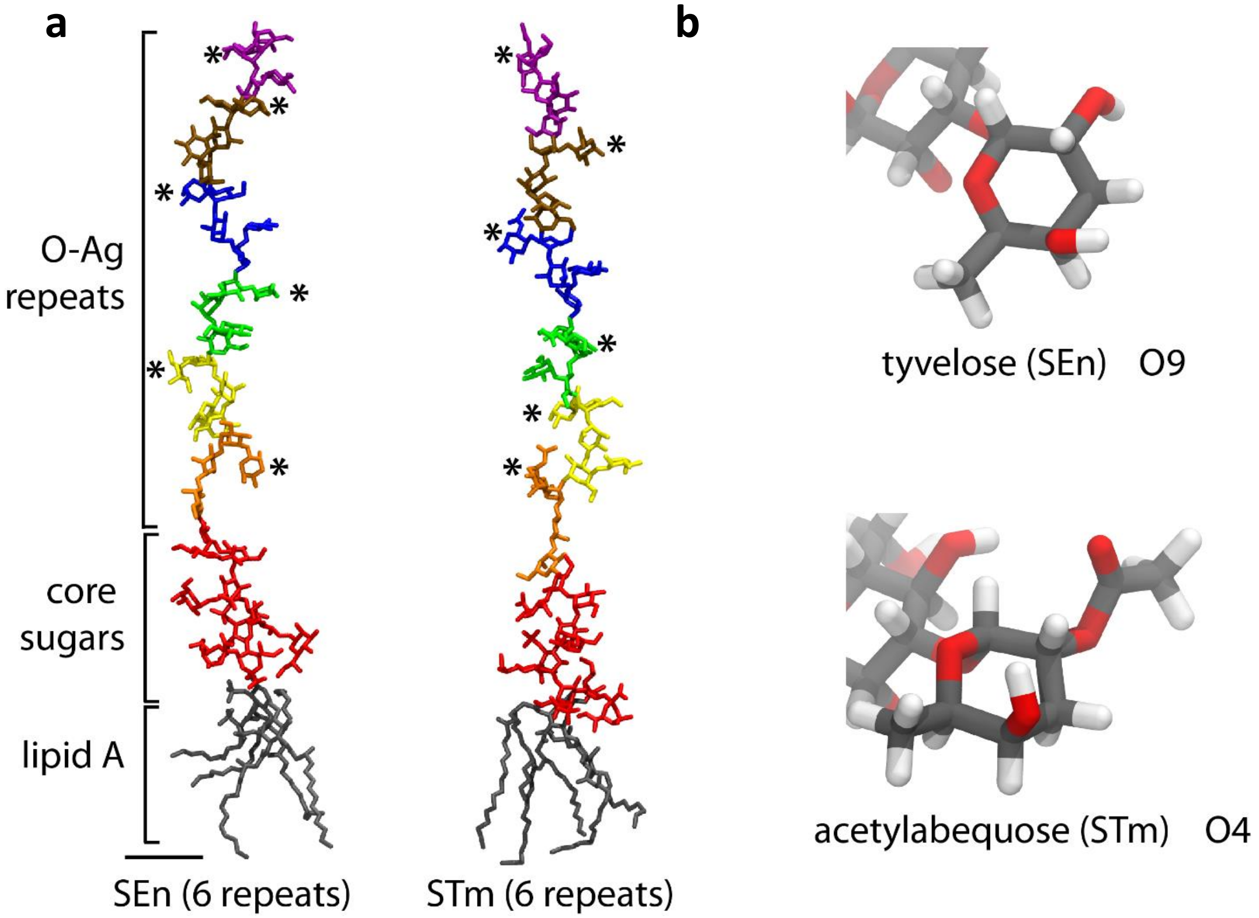

Supplementary Fig. 3. Differences between SEn O-Ag and STm O-Ag. **a** Comparison between two example O-Ag chains, each with six repeats. Lipid A (black) and the core sugars (red) are also shown. The asterisks indicate the position of the unique sugars, tyvelose and acetylabequose. LPS elements are coloured as in Fig. 4B. **b** The difference between the O-Ag structure of the two serovars is the presence of tyvelose in the O-Ag repeat of SEn (top) and acetylabequose in that of STm (bottom).

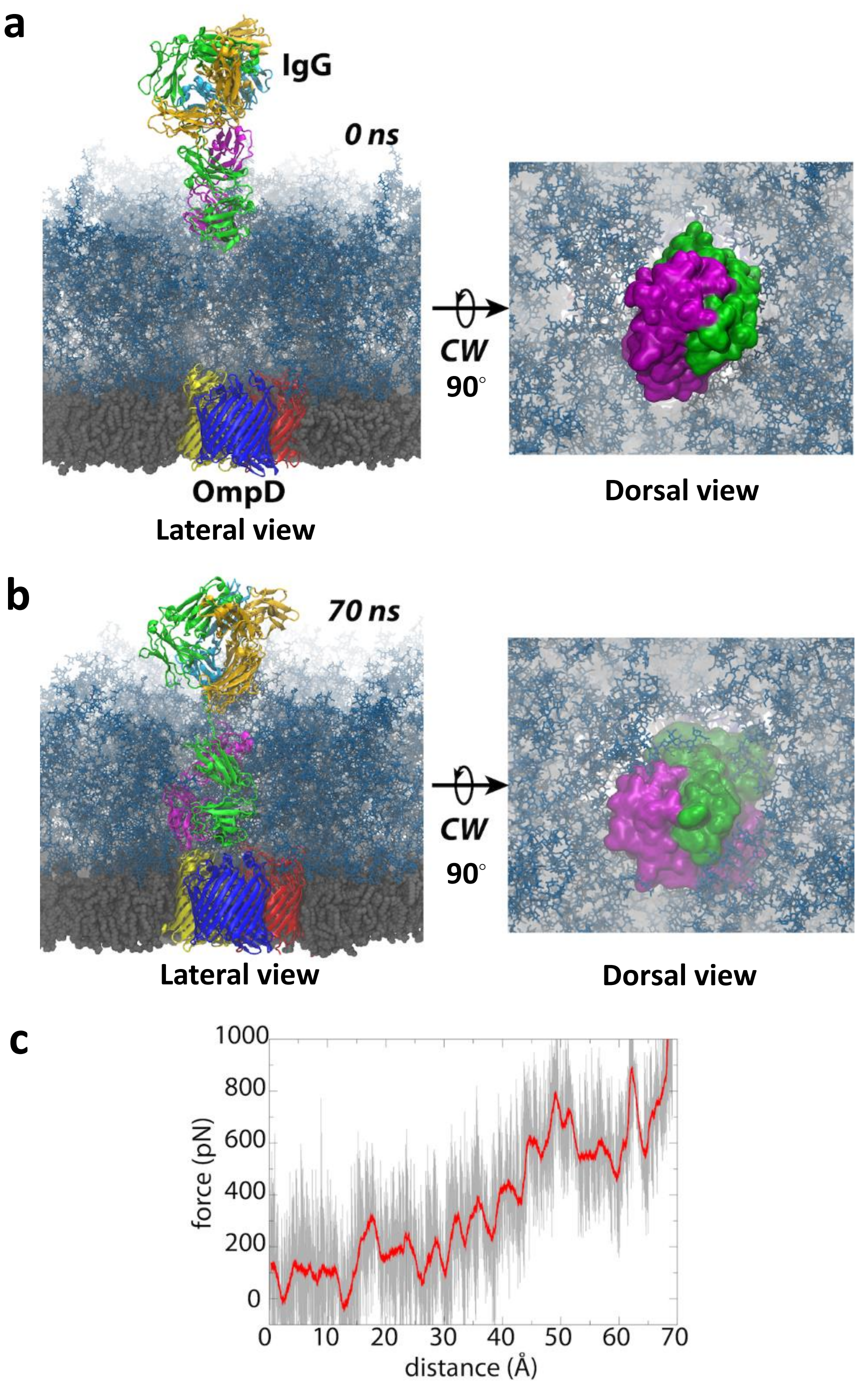

Supplementary Fig. 4. Steered MD simulation of IgG-OmpD interactions. This demonstrates that surface epitopes are accessible by a single Fab. The proteins are colored as in Fig. 4. **a** Initial state of the system with the antibody positioned at the edge of the O-Ag viewed from the membrane plane (left) and from the extracellular side (right). **b** Final state of the system after 70 ns of SMD at 1.0 Å/ns. One Fab of IgG is in contact with OmpD. **c** Force vs. distance for the SMD simulation. The force gradually rises as the Fab penetrates deeper into the O-Ag, peaking as it encounters OmpD. CW = Clockwise.

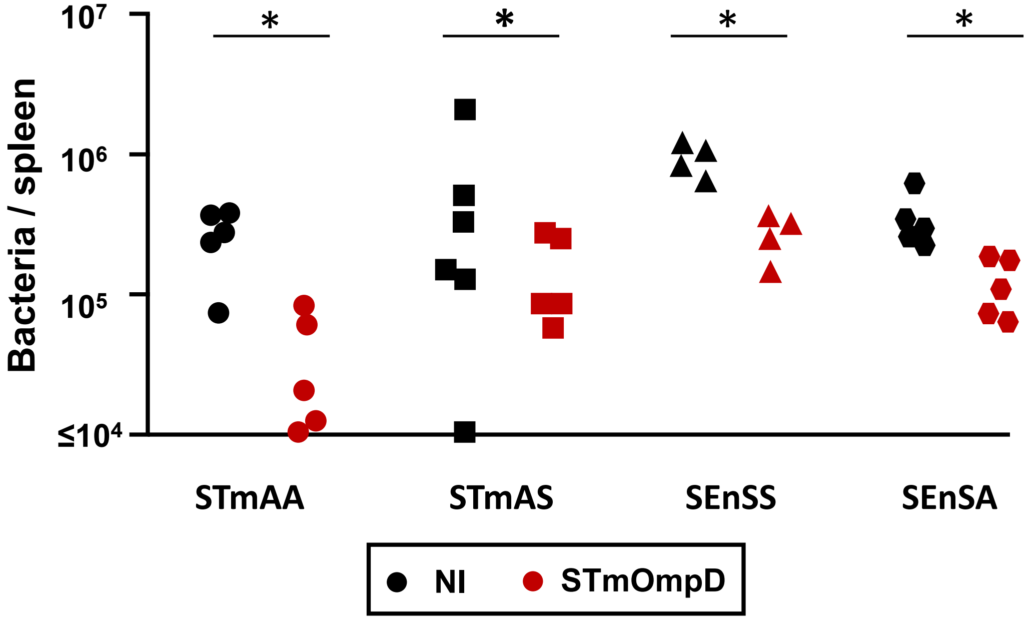

Supplementary Fig. 5. Challenge with OmpD mutant bacteria. Matching native OmpD and O-Ag are required for efficient protection by STmOmpD antibodies. Splenic bacterial numbers of NI WT mice and WT mice immunized twice with 20 µg STmOmpD on days 0 and 14 and challenged 7 days later with virulent STm or SEn mutants that express WT OmpD (STmAA and SEnSS respectively) or STm or SEn mutated to express the heterologous OmpD amino acid at residue 263 (i.e. A→S for STm and S→A for SEn; STmAS and SEnSA respectively) for 24 hours. * = $P \leq 0.05$, ** = $P \leq 0.01$ assessed by 2-tailed Mann-Whitney U-test.

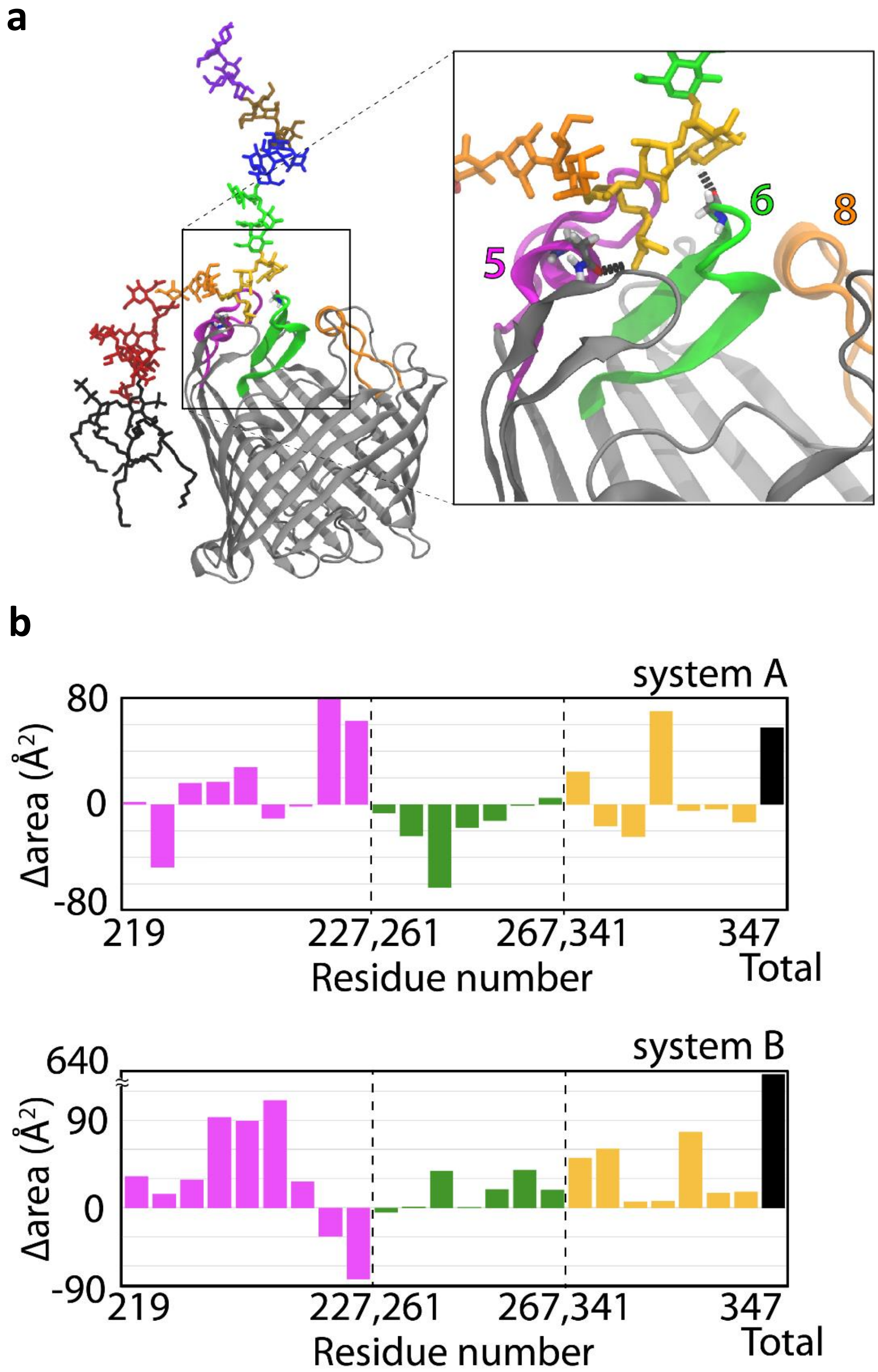

Supplementary Fig. 6. Interactions between O-Ag and OmpD. **a** Snapshot from the MD simulation of SEn OmpD and the outer membrane showing one O-Ag chain forming hydrogen bonds with extracellular loops 5 and 6 (inset). Other extracellular loops colored as per Fig. 4 and Fig. S2. **b** Difference in interaction area between SEn and STm OmpD and O-Ag for both systems (A and B), colored by loop (black is total). Areas are summed over the three protomers and averaged over two 500-ns runs initiated after 1 μs of equilibration for each system (two SEn and two STm systems). A number greater than zero indicates greater occlusion by OAg in SEn than in STm.

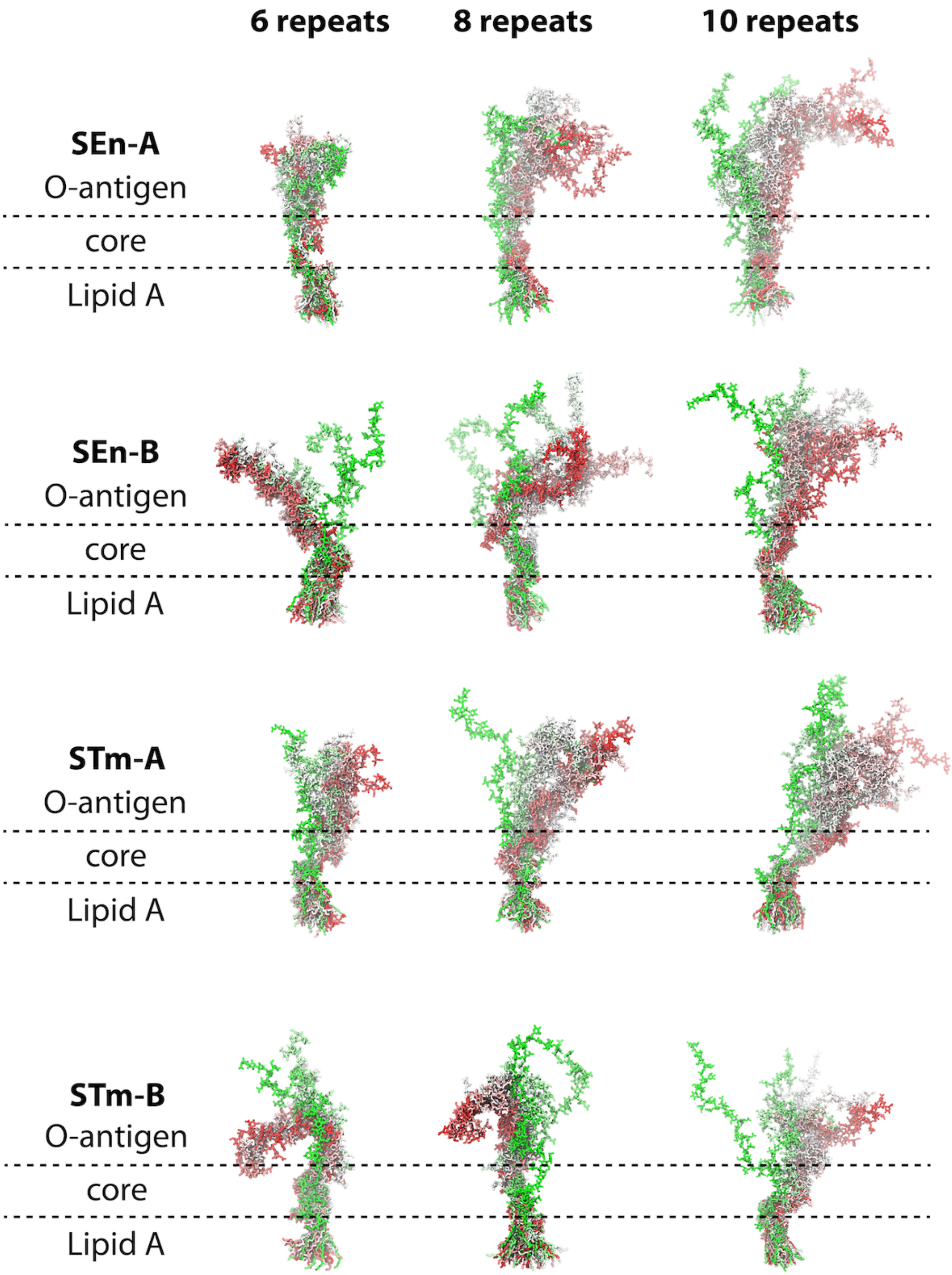

Supplementary Fig. 7. Motion of selected O-Ag chains. This is shown for each of the four MD systems, SEn (A and B) and STm (A and B). For each system, 11 snapshots, each of three randomly selected chains of the indicated length (6, 8, and 10 repeats), are overlaid. In each image, green represents the initial conformation of the O-Ag, white that after 750 ns, and red that after 1.5 μs.

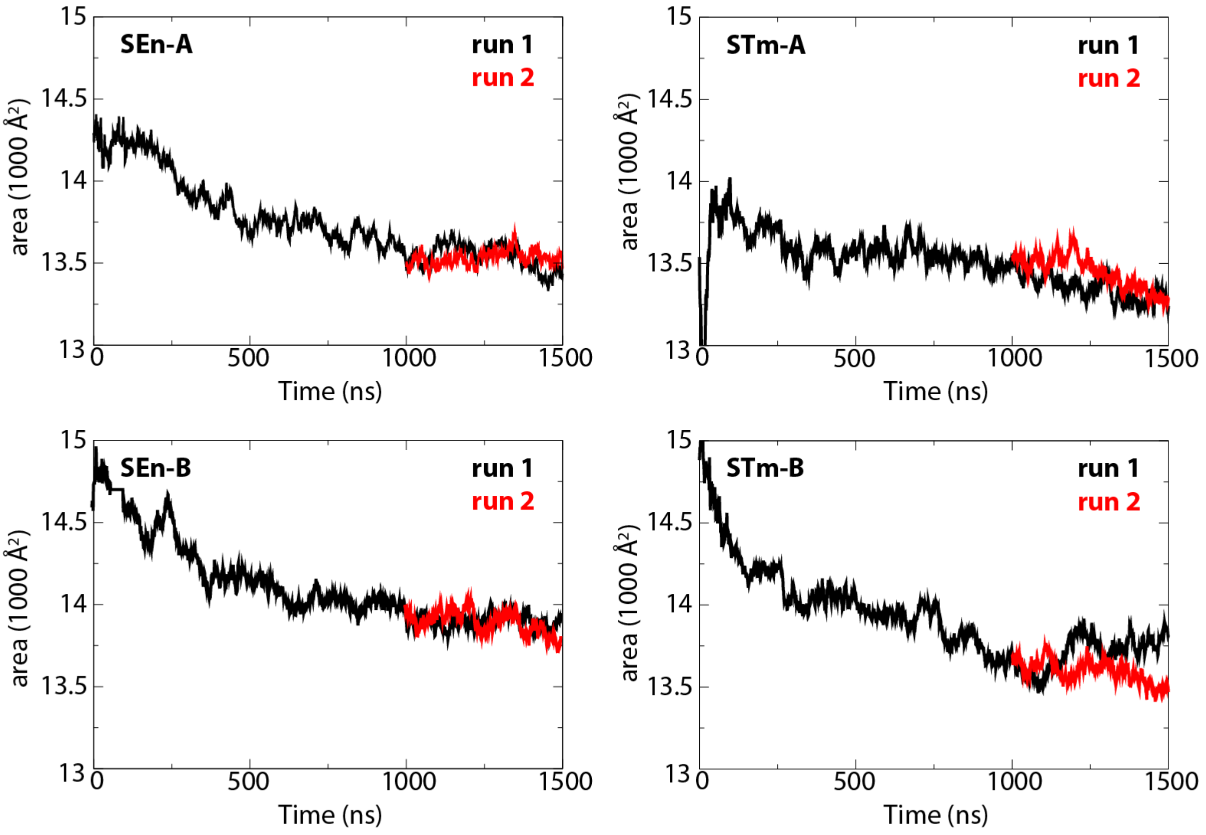

Supplementary Fig. 8. Membrane area vs. time. This is shown for each of the four MD systems, SEn (A and B) and STm (A and B). After 1 μs, each simulation was replicated, indicated by the additional red curve for the second run.

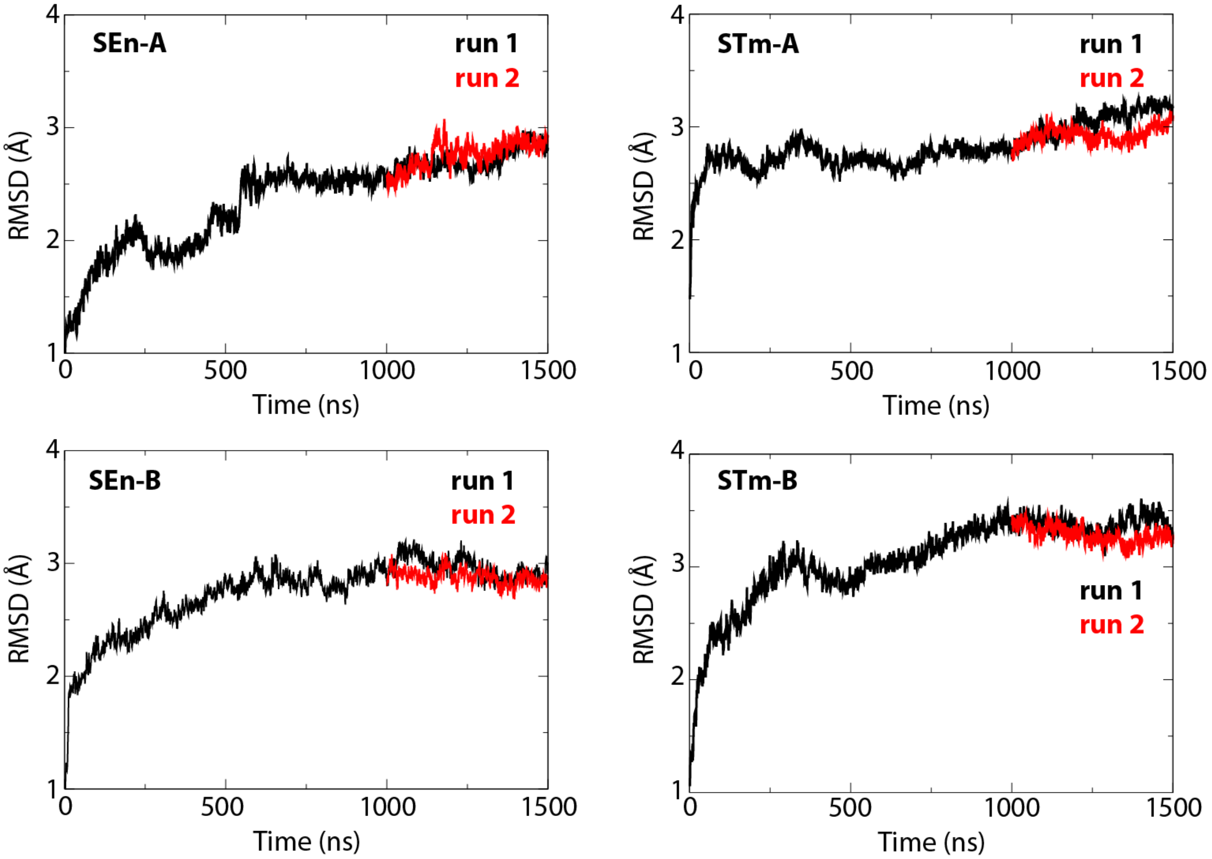

Supplementary Fig. 9. Root mean-square deviation (RMSD). This is shown for the OmpD trimer (backbone only) for each of the four MD systems, SEn (A and B) and STm (A and B). After 1 µs, each simulation was replicated, indicated by the additional red curve for the second run.
